# Ethyl (3*S*)-3-[(3a*R*,5*R*,6*S*,6a*R*)-6-hy­droxy-2,2-di­methyl­tetra­hydro­furo[4,5-*d*][1,3]dioxol-5-yl]-3-{(3*S*)-3-[(3a*R*,5*R*,6*S*,6a*R*)-6-hy­droxy-2,2-di­methyl­tetra­hydro­furo[4,5-*d*][1,3]dioxol-5-yl]-5-oxoisoxazolidin-2-yl}propano­ate chloro­form monosolvate

**DOI:** 10.1107/S2414314620007889

**Published:** 2020-06-16

**Authors:** Aldo Guillermo Amaro Hernández, Tomasa Rodríguez Tzompantzi, Álvaro Dávila García, Rosa Luisa Meza-León, Sylvain Bernès

**Affiliations:** a Facultad de Ciencias Químicas, Benemérita Universidad Autónoma de Puebla, 14 Sur Esq. Av. San Claudio, 72570 Puebla, Pue., Mexico; b Instituto de Física, Benemérita Universidad Autónoma de Puebla, Av. San Claudio y 18 Sur, 72570 Puebla, Pue., Mexico; University of Antofagasta, Chile

**Keywords:** crystal structure, solvate, absolute structure, Flack parameter, 2*AD* plot

## Abstract

The title compound, C_22_H_33_NO_12_·CHCl_3_, was obtained as a product of a double aza-Michael addition of hydroxyl­amine on a Chiron with a known absolute configuration. The enanti­opure compound crystallized as a chloro­form solvate, in space group *P*1, and diffraction data were collected at room temperature with Ag *K*α radiation. The Flack parameter refined to *x* = −0.01 (16); however, the Flack and Watkin 2*AD* plot clearly shows that differences between Friedel opposites (the *D* component of the plot) do not carry any reliable information about resonant scattering of Cl atoms, and are rather dominated by random and systematic errors. The *R_D_* factor calculated using 1941 acentric Friedel pairs is *R_D_* = 0.995. On the other hand, the 2*A* component of the plot, related to average intensities of Friedel pairs, shows that data are of good quality (*R_A_* = 0.069). This example illustrates that while using Ag *K*α radiation (λ = 0.56083 Å), scatterers heavier than Cl should be present in a chiral crystal in order to determine confidently the absolute structure of the crystal.

## Abstract 2

The Flack and Watkin 2*AD* plot based on 1941 acentric Friedel pairs for the title chloro­form solvate shows that the observed intensity differences for Friedel opposites are dominated by random and systematic errors, erasing information about resonant scattering.

## Graphical abstract

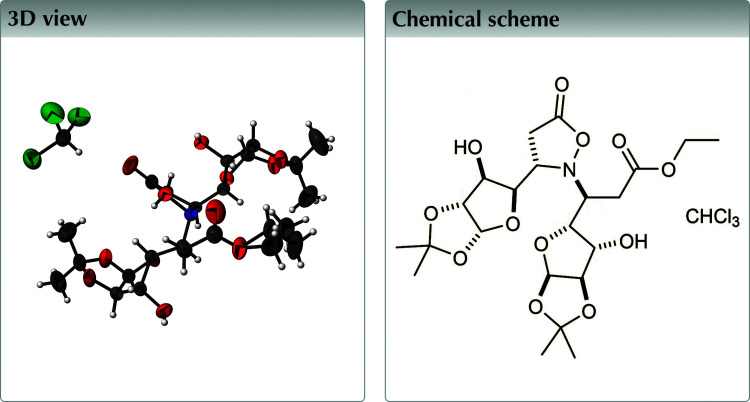

## Structure description

The Chiron known as 7,3-LXF (7,3-lactone-xylo­furan­ose derivative; Ramírez *et al.*, 2017 ▸), derived from d-glucose, is a versatile starting material for the synthesis of natural products, for example the metabolites produced by *Trichoderma* spp and *Penicillium* isolates (Pérez-Bautista *et al.*, 2016 ▸). In a work aimed at the synthesis of 1-de­oxy­nojirimycin (DNJ), an aza­sugar alkaloid presenting α-glucosidase inhibitor properties, the title compound was obtained (Amaro Hernández, 2019 ▸). The total synthesis of DNJ has been reported, for example starting from d-glucose (Khobare *et al.*, 2016 ▸). However, the stereochemistry of 7,3-LXF matches the stereochemistry of the target mol­ecule, and 7,3-LXF is thus considered to be an ideal Chiron for the synthesis of DNJ. Moreover, we developed an efficient procedure for the preparation of 7,3-LXF at the gram scale.

The title compound was obtained while attempting an aza-Michael addition of hydroxyl­amine to 7,3-LXF, at pH 7. Under our experimental conditions, a double aza-Michael addition was observed, followed by a transesterification in ethanol, affording a disubstituted isoxazolidinone, which was characterized by X-ray diffraction. This compound is also closely related to other isoxazolidinone derivatives obtained through an Amadori rearrangement, which were studied for their potential anti­oxidant properties, and their application as flood flavouring agents (Hodge, 1955 ▸; Mills & Hodge, 1976 ▸; Mills, 1979 ▸).

**Figure 1:**
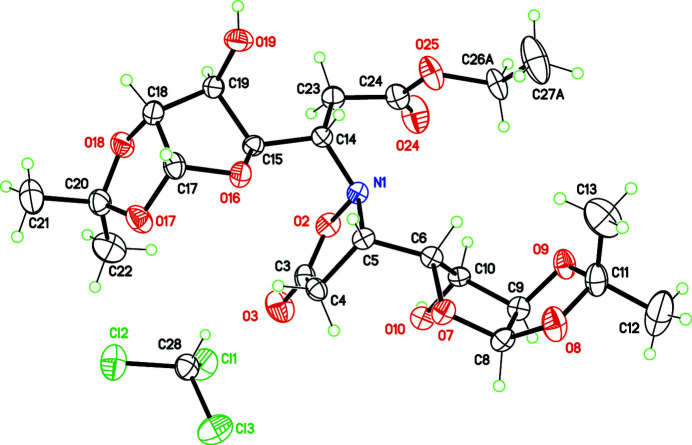
Structure of the title compound, with displacement ellipsoids for non-H atoms at the 30% probability level. For the disordered ethyl group in the ester functionality, only one disordered position is retained [*A* site, with occupancy of 0.58 (5)].

The enanti­opure mol­ecule was crystallized as a chloro­form solvate, in space group *P*1 (Fig. 1 ▸). The core isoxazolidinone ring has the expected envelope conformation, with C5 as the flap. The ring is, however, close to being flat, with a puckering parameter *q*_2_ = 0.190 (5) Å. The ring is substituted at C5 and N1 by the bicyclic groups provided by the Chiron. The absolute configuration at C5 is imposed as 5*S*, while the stereochemistry at N1 is not imposed by the Michael addition. Substituents at C5 and N1 are thus arranged *trans* with respect to the isoxazolidinone plane, avoiding in this way any steric hindrance. In the crystal structure, only weak inter­molecular O—H⋯O hydrogen bonds are formed, involving hy­droxy groups O10 and O19 (Table 1 ▸). The chloro­form lattice mol­ecule does not inter­act with the organic mol­ecule.

**Figure 2:**
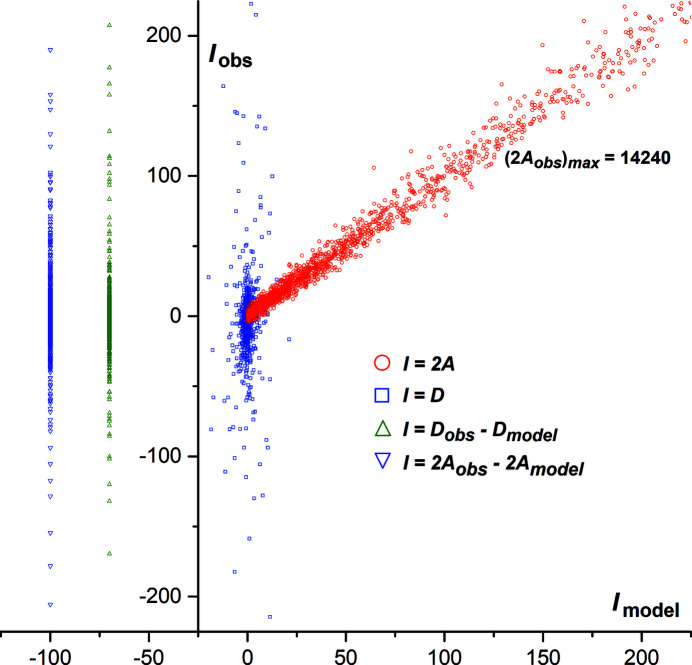
2*AD* plot for 1941 acentric Friedel pairs retrieved from the *SHELXL fcf* file for the last refinement cycle of the title compound (Sheldrick, 2015*b* ▸). The *D*_obs_ against *D*_model_ of all Friedel pairs (blue squares) and the 2*A*_obs_ against 2*A*_model_ for weak Friedel pairs (red circles) are displayed. On the left, *D*_obs_ − *D*_model_ (green triangles) and 2*A*_obs_ − 2*A*_model_ (violet triangles) of all Friedel pairs are displayed, at arbitrary fixed abscissa. The style of the 2*AD* plot follows that used in the articles of Flack *et al.* (see, for example, Fig. 3 in Parsons *et al.*, 2012 ▸).

For this Cl-containing crystal, intensities were collected at room temperature using Ag *K*α radiation. With such an experimental setup, the refined Flack (1983 ▸) parameter converges to *x* = −0.01 (16) for the correct absolute structure, and *x* = 0.85 (16) for the inverted structure, giving the false impression that chlorine anomalous dispersion allows the reliable determination of the absolute configuration for the mol­ecule. Similar metrics are obtained using the Parsons intensity quotients method (Parsons *et al.*, 2013 ▸), or by refining the structure as an inversion twin (Sheldrick, 2015*b* ▸). However, the 2*AD* graphs devised by David Watkin and Howard Flack are a valuable tool for estimating whether real information about resonant scattering is present in the measured intensities (Flack *et al.*, 2011 ▸; Parsons *et al.*, 2012 ▸). The average (*A*) and difference (*D*) intensities for Friedel opposites are defined by *A*(**h**) = ½[|F(**h**)|^2^ + |F(−**h**)|^2^] and *D*(**h**) = |F(**h**)|^2^ − |F(−**h**)|^2^. In a 2*AD* graph, *D*_obs_ against *D*_model_ of the acentric reflections is plotted, as well as 2*A*_obs_ against 2*A*_model_ for weak reflections. For the 2*A* plot, a distribution of points spread around a straight line of slope 1 passing through the origin indicates that diffraction data are of good quality, and this is indeed the case for the title compound (Fig. 2 ▸). The *D* plot is much more instructive regarding the accuracy of data for measuring anomalous dispersion: the greater the slope of this distribution deviates from 1, the more the effects of anomalous dispersion are overwhelmed by random uncertainty and systematic errors. This is clearly the case for the title compound, despite the presence of three Cl atoms in the asymmetric unit: for the *D* distribution, all data points are placed close to *D*_model_ = 0 on the *D*_obs_ axis, as is the case for any centrosymmetric structure (Fig. 2 ▸). Classical *R* unweighted factors can also be computed for *A* and *D*, which reflect the deviation from the unity-slope distribution: *R_A_* = Σ|*A*_obs_(**h**) − *A*_model_(**h**)|/Σ|*A*_obs_(**h**)| and *R_D_* = Σ|*D*_obs_(**h**) − *D*_model_(**h**)|/Σ|*D*_obs_(**h**)|, where the summations are over paired acentric reflections **h** and −**h** (note that in space group *P*1, all reflections are acentric, and that *R*_A_ is then conceptually close to *R*_int_). For the title compound, *R_A_* = 0.069 and *R_D_* = 0.995. The large *R_D_* factor is obviously in line with the large standard uncertainty of the refined Flack parameter, *u*(*x*) = 0.16. In the crystal studied here, undue reliance should not be placed on the Flack parameter, and the absolute configuration of the mol­ecule should instead be assigned by relying on the chemistry.

In conclusion, we have shown that a CHCl_3_ mol­ecule is certainly not sufficient for determining the absolute structure of a chiral crystal if Ag *K*α radiation is used for collecting intensities. On a broader front, it is worth reminding that the standard uncertainty in the Flack parameter, *u*(*x*), is the key to its correct inter­pretation (Flack & Bernardinelli, 2000 ▸; Thompson & Watkin, 2009 ▸). The use of 2*AD* plots is thus strongly advised for the validation of absolute-structure determinations (Flack, 2012 ▸), together with Flack *x* and Hooft *y* parameters. Unfortunately, these plots are not yet used on a routine basis in chemical crystallography.

## Synthesis and crystallization

A solution of NH_2_OH·HCl (85 mg, 0.025 mmol) in water (1 ml) was neutralized with a solution of NaHCO_3_ (pH 7). After 10 min., a solution of 7,3-LXF (50 mg, 0.23 mmol) in ethanol (3 ml) was added over 30 s. and the mixture was left under stirring at room temperature. The reaction was complete after one h. The mixture was filtered over celite/Na_2_SO_4_, and the filtrate was reduced to give yellow solids, which were purified by column chromatography (hexa­ne:ethyl acetate, 1:1), to afford 95 mg of the title compound (yield: 80%). Colourless single crystals were obtained by slow evaporation of a MeOH/CHCl_3_ solution.

## Refinement

Crystal data, data collection and structure refinement details are summarized in Table 2 ▸. The ethyl group C26–C27 is disordered over two positions, C26*A*/C27*A* [occupancy: 0.58 (5)] and C26*B*/C27*B* [occupancy: 0.42 (5)].

## Supplementary Material

Crystal structure: contains datablock(s) I, global. DOI: 10.1107/S2414314620007889/bx4018sup1.cif

Structure factors: contains datablock(s) I. DOI: 10.1107/S2414314620007889/bx4018Isup2.hkl

Supporting information file. DOI: 10.1107/S2414314620007889/bx4018Isup3.cml

CCDC reference: 2009153

Additional supporting information:  crystallographic information; 3D view; checkCIF report

## Figures and Tables

**Table 1:** Hydrogen-bond geometry (Å, °)

*D*—H⋯*A*	*D*—H	H⋯*A*	*D*⋯*A*	*D*—H⋯*A*
O10—H10⋯O7^i^	0.85 (1)	1.94 (2)	2.778 (4)	169 (6)
O19—H19⋯O10^ii^	0.85 (1)	1.98 (3)	2.798 (5)	162 (7)

Symmetry codes: (i) 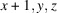 ; (ii) 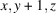 .

**Table 2:** Experimental details

Crystal data	
Chemical formula	C_22_H_33_NO_12_·CHCl_3_
*M*_r_	622.86
Crystal system, space group	Triclinic, *P*1
Temperature (K)	296
*a*, *b*, *c* (Å)	5.5734 (4), 9.2537 (9), 14.2547 (12)
α, β, γ (°)	91.995 (7), 99.103 (6), 95.567 (7)
*V* (Å^3^)	721.56 (11)
*Z*	1
Radiation type	Ag *K*α, λ = 0.56083 Å
μ (mm^−1^)	0.20
Crystal size (mm)	0.37 × 0.35 × 0.15

Data collection	
Diffractometer	Stoe Stadivari
Absorption correction	Multi-scan (*X-AREA*; Stoe & Cie, 2018 ▸)
*T*_min_, *T*_max_	0.435, 1.000
No. of measured, independent and observed [*I* > 2σ(*I*)] reflections	14451, 4682, 3696
*R*_int_	0.038
(sin θ/λ)_max_ (Å^−1^)	0.610

Refinement	
*R*[*F* ^2^ > 2σ(*F* ^2^)], *wR*(*F* ^2^), *S*	0.048, 0.134, 1.01
No. of reflections	4682
No. of parameters	383
No. of restraints	5
H-atom treatment	H atoms treated by a mixture of independent and constrained refinement
Δρ_max_, Δρ_min_ (e Å^−3^)	0.30, −0.24

Computer programs: *X-AREA* (Stoe & Cie, 2018 ▸), *SHELXT2014/5* (Sheldrick, 2015*a* ▸), *SHELXL2018/1* (Sheldrick, 2015*b* ▸), *XP* in *SHELXTL-Plus* (Sheldrick, 2008 ▸), *Mercury* (Macrae *et al.*, 2020 ▸) and *publCIF* (Westrip, 2010 ▸).

## full crystallographic data

### Crystal data

**Table:**

C_22_H_33_NO_12_·CHCl_3_	*F*(000) = 326
*M**_r_* = 622.86	*D*_x_ = 1.433 Mg m^−^^3^
Triclinic, *P*1	Melting point: 472 K
*a* = 5.5734 (4) Å	Ag *K*α radiation, λ = 0.56083 Å
*b* = 9.2537 (9) Å	Cell parameters from 12914 reflections
*c* = 14.2547 (12) Å	θ = 2.8–22.1°
α = 91.995 (7)°	µ = 0.20 mm^−^^1^
β = 99.103 (6)°	*T* = 296 K
γ = 95.567 (7)°	Prism, colourless
*V* = 721.56 (11) Å^3^	0.37 × 0.35 × 0.15 mm
*Z* = 1	

### Data collection

**Table:**

Stoe Stadivari diffractometer	4682 independent reflections
Radiation source: Sealed X-ray tube, Axo Astix-f Microfocus source	3696 reflections with *I* > 2σ(*I*)
Graded multilayer mirror monochromator	*R*_int_ = 0.038
Detector resolution: 5.81 pixels mm^-1^	θ_max_ = 20.0°, θ_min_ = 2.8°
ω scans	*h* = −6→6
Absorption correction: multi-scan (X-AREA; Stoe & Cie, 2018)	*k* = −11→11
*T*_min_ = 0.435, *T*_max_ = 1.000	*l* = −17→17
14451 measured reflections	

### Refinement

**Table:**

Refinement on *F*^2^	Primary atom site location: dual
Least-squares matrix: full	Secondary atom site location: difference Fourier map
*R*[*F*^2^ > 2σ(*F*^2^)] = 0.048	Hydrogen site location: mixed
*wR*(*F*^2^) = 0.134	H atoms treated by a mixture of independent and constrained refinement
*S* = 1.00	*w* = 1/[σ^2^(*F*_o_^2^) + (0.0893*P*)^2^] where *P* = (*F*_o_^2^ + 2*F*_c_^2^)/3
4682 reflections	(Δ/σ)_max_ < 0.001
383 parameters	Δρ_max_ = 0.30 e Å^−^^3^
5 restraints	Δρ_min_ = −0.24 e Å^−^^3^
0 constraints	

### Special details

**Table:**

Refinement. H atoms bonded to C atoms were placed in calculated positions and the hydroxy H atoms H10/H19 were refined with free coordinates and O—H bond lengths restrained to 0.85 (1) Å.

### Fractional atomic coordinates and isotropic or equivalent isotropic displacement parameters (Å^2^)

**Table:**

	*x*	*y*	*z*	*U*_iso_*/*U*_eq_	Occ. (<1)
N1	0.4192 (6)	0.3430 (4)	0.5695 (3)	0.0344 (8)	
O2	0.5788 (5)	0.2914 (4)	0.5055 (2)	0.0427 (8)	
C3	0.4543 (9)	0.1909 (6)	0.4400 (3)	0.0439 (11)	
O3	0.5583 (9)	0.1395 (6)	0.3811 (3)	0.0753 (13)	
C4	0.1949 (8)	0.1629 (5)	0.4547 (3)	0.0385 (10)	
H4A	0.146436	0.059472	0.455717	0.046*	
H4B	0.085767	0.203684	0.405008	0.046*	
C5	0.1936 (7)	0.2394 (5)	0.5516 (3)	0.0325 (9)	
H5A	0.049758	0.293233	0.548463	0.039*	
C6	0.2096 (7)	0.1456 (5)	0.6367 (3)	0.0325 (9)	
H6A	0.182642	0.205556	0.691308	0.039*	
O7	0.0169 (5)	0.0254 (3)	0.6205 (2)	0.0383 (7)	
C8	0.0764 (8)	−0.0827 (5)	0.6869 (4)	0.0401 (10)	
H8A	0.051856	−0.180065	0.655473	0.048*	
O8	−0.0546 (6)	−0.0761 (5)	0.7624 (3)	0.0591 (11)	
C9	0.3446 (8)	−0.0429 (5)	0.7322 (3)	0.0395 (10)	
H9A	0.438964	−0.127142	0.737062	0.047*	
O9	0.3312 (6)	0.0235 (4)	0.8219 (2)	0.0507 (9)	
C10	0.4372 (7)	0.0710 (5)	0.6671 (3)	0.0331 (9)	
H10A	0.570310	0.138973	0.701591	0.040*	
O10	0.5094 (5)	−0.0006 (4)	0.5884 (2)	0.0392 (7)	
H10	0.663 (3)	0.018 (7)	0.595 (4)	0.059*	
C11	0.0999 (10)	−0.0210 (7)	0.8482 (4)	0.0556 (14)	
C12	0.1196 (18)	−0.1414 (13)	0.9156 (7)	0.110 (3)	
H12A	0.153220	−0.227703	0.882906	0.164*	
H12B	−0.031500	−0.159948	0.939536	0.164*	
H12C	0.249583	−0.113622	0.967635	0.164*	
C13	−0.0003 (15)	0.1123 (11)	0.8849 (6)	0.092 (2)	
H13A	−0.003399	0.184682	0.838227	0.138*	
H13B	0.101958	0.150507	0.942771	0.138*	
H13C	−0.163125	0.086339	0.897110	0.138*	
C14	0.3958 (8)	0.4978 (5)	0.5519 (3)	0.0341 (9)	
H14A	0.275126	0.530272	0.588829	0.041*	
C15	0.3155 (8)	0.5318 (5)	0.4486 (3)	0.0358 (10)	
H15A	0.417651	0.485722	0.408769	0.043*	
O16	0.0651 (6)	0.4747 (4)	0.4186 (2)	0.0431 (8)	
C17	−0.0249 (9)	0.5423 (6)	0.3350 (3)	0.0431 (11)	
H17A	−0.190930	0.567791	0.336470	0.052*	
O17	−0.0169 (7)	0.4560 (4)	0.2531 (3)	0.0559 (10)	
C18	0.1501 (10)	0.6781 (6)	0.3303 (3)	0.0458 (11)	
H18A	0.065765	0.764689	0.315529	0.055*	
O18	0.2886 (7)	0.6389 (4)	0.2598 (3)	0.0537 (9)	
C19	0.3149 (9)	0.6928 (5)	0.4266 (3)	0.0416 (11)	
H19A	0.478703	0.739458	0.423355	0.050*	
O19	0.1960 (8)	0.7677 (4)	0.4926 (3)	0.0553 (9)	
H19	0.285 (12)	0.846 (5)	0.510 (5)	0.083*	
C20	0.1496 (10)	0.5257 (6)	0.1982 (4)	0.0501 (12)	
C21	0.0011 (17)	0.5882 (9)	0.1133 (5)	0.088 (2)	
H21A	−0.096745	0.658066	0.135300	0.132*	
H21B	−0.103333	0.511294	0.076155	0.132*	
H21C	0.109420	0.634776	0.074853	0.132*	
C22	0.3192 (14)	0.4193 (9)	0.1729 (5)	0.0780 (19)	
H22A	0.398296	0.380374	0.229878	0.117*	
H22B	0.440117	0.467836	0.140609	0.117*	
H22C	0.227581	0.341646	0.132050	0.117*	
C23	0.6434 (9)	0.5822 (6)	0.5916 (4)	0.0448 (11)	
H23A	0.758166	0.567412	0.548500	0.054*	
H23B	0.625021	0.685251	0.596046	0.054*	
C24	0.7432 (9)	0.5331 (6)	0.6887 (4)	0.0493 (12)	
O24	0.9310 (8)	0.4794 (7)	0.7079 (3)	0.0849 (15)	
O25	0.5936 (8)	0.5557 (6)	0.7498 (3)	0.0674 (12)	
C26A	0.658 (3)	0.478 (3)	0.8403 (10)	0.057 (6)	0.58 (5)
H26A	0.664087	0.375099	0.825648	0.069*	0.58 (5)
H26B	0.816300	0.517989	0.874147	0.069*	0.58 (5)
C27A	0.462 (4)	0.498 (5)	0.9003 (15)	0.104 (11)	0.58 (5)
H27A	0.481627	0.435909	0.952970	0.156*	0.58 (5)
H27B	0.304371	0.473354	0.862377	0.156*	0.58 (5)
H27C	0.476913	0.597468	0.923689	0.156*	0.58 (5)
C26B	0.640 (7)	0.552 (5)	0.850 (2)	0.086 (9)	0.42 (5)
H26C	0.593631	0.639463	0.879415	0.104*	0.42 (5)
H26D	0.811343	0.544653	0.872685	0.104*	0.42 (5)
C27B	0.492 (7)	0.426 (3)	0.872 (2)	0.079 (9)	0.42 (5)
H27D	0.554422	0.397414	0.934526	0.119*	0.42 (5)
H27E	0.497246	0.348130	0.826281	0.119*	0.42 (5)
H27F	0.326809	0.448081	0.869704	0.119*	0.42 (5)
C28	0.7434 (11)	0.0620 (7)	0.1880 (4)	0.0593 (14)	
H28A	0.720244	0.127719	0.240495	0.071*	
Cl1	1.0534 (3)	0.0408 (2)	0.19859 (17)	0.0881 (6)	
Cl2	0.6390 (4)	0.1396 (3)	0.08089 (14)	0.0907 (6)	
Cl3	0.5806 (3)	−0.1062 (2)	0.19867 (17)	0.0876 (6)	

### Atomic displacement parameters (Å^2^)

**Table:**

	*U*^11^	*U*^22^	*U*^33^	*U*^12^	*U*^13^	*U*^23^
N1	0.0274 (16)	0.038 (2)	0.0385 (19)	0.0029 (14)	0.0079 (14)	0.0036 (15)
O2	0.0276 (15)	0.0475 (19)	0.057 (2)	0.0075 (14)	0.0166 (14)	0.0068 (16)
C3	0.042 (2)	0.055 (3)	0.039 (3)	0.013 (2)	0.012 (2)	0.008 (2)
O3	0.077 (3)	0.099 (4)	0.060 (3)	0.027 (3)	0.034 (2)	−0.007 (2)
C4	0.036 (2)	0.045 (3)	0.034 (2)	0.0087 (19)	0.0007 (18)	−0.0011 (19)
C5	0.0218 (18)	0.035 (2)	0.042 (2)	0.0052 (16)	0.0053 (16)	0.0030 (18)
C6	0.0201 (18)	0.038 (2)	0.038 (2)	0.0008 (16)	0.0049 (16)	−0.0001 (18)
O7	0.0206 (14)	0.0443 (18)	0.0483 (18)	−0.0026 (12)	0.0028 (12)	0.0098 (14)
C8	0.029 (2)	0.040 (3)	0.050 (3)	−0.0023 (18)	0.0055 (18)	0.007 (2)
O8	0.0375 (18)	0.093 (3)	0.047 (2)	−0.0056 (19)	0.0126 (15)	0.011 (2)
C9	0.032 (2)	0.045 (3)	0.043 (3)	0.0077 (19)	0.0056 (18)	0.005 (2)
O9	0.0381 (17)	0.074 (2)	0.0380 (19)	0.0017 (17)	0.0033 (14)	0.0078 (17)
C10	0.0237 (19)	0.033 (2)	0.041 (2)	−0.0010 (16)	0.0028 (16)	−0.0006 (18)
O10	0.0225 (13)	0.0476 (19)	0.0476 (18)	0.0058 (13)	0.0057 (12)	−0.0001 (15)
C11	0.043 (3)	0.081 (4)	0.045 (3)	0.001 (3)	0.011 (2)	0.016 (3)
C12	0.092 (6)	0.139 (8)	0.088 (6)	−0.026 (5)	−0.004 (4)	0.057 (6)
C13	0.067 (4)	0.127 (7)	0.088 (5)	0.018 (4)	0.032 (4)	−0.016 (5)
C14	0.0297 (19)	0.036 (2)	0.036 (2)	−0.0022 (17)	0.0063 (16)	0.0001 (18)
C15	0.035 (2)	0.034 (2)	0.038 (3)	−0.0013 (18)	0.0085 (18)	0.0020 (19)
O16	0.0374 (17)	0.0467 (19)	0.0411 (18)	−0.0065 (14)	−0.0014 (14)	0.0110 (15)
C17	0.039 (2)	0.054 (3)	0.037 (2)	0.011 (2)	0.0043 (19)	0.005 (2)
O17	0.058 (2)	0.067 (2)	0.041 (2)	−0.0131 (19)	0.0138 (16)	−0.0052 (17)
C18	0.059 (3)	0.043 (3)	0.038 (3)	0.011 (2)	0.009 (2)	0.007 (2)
O18	0.068 (2)	0.054 (2)	0.0394 (19)	−0.0106 (18)	0.0182 (16)	0.0038 (16)
C19	0.051 (3)	0.038 (2)	0.037 (3)	0.002 (2)	0.011 (2)	0.004 (2)
O19	0.065 (2)	0.043 (2)	0.058 (2)	0.0053 (17)	0.0159 (19)	−0.0085 (17)
C20	0.054 (3)	0.056 (3)	0.039 (3)	−0.004 (2)	0.011 (2)	0.007 (2)
C21	0.116 (7)	0.092 (5)	0.049 (4)	0.001 (5)	−0.004 (4)	0.016 (4)
C22	0.068 (4)	0.089 (5)	0.080 (5)	0.006 (4)	0.027 (4)	−0.012 (4)
C23	0.038 (2)	0.047 (3)	0.046 (3)	−0.008 (2)	0.002 (2)	0.002 (2)
C24	0.036 (3)	0.060 (3)	0.048 (3)	−0.004 (2)	0.002 (2)	0.000 (2)
O24	0.047 (2)	0.138 (5)	0.070 (3)	0.023 (3)	0.000 (2)	0.017 (3)
O25	0.053 (2)	0.107 (4)	0.040 (2)	0.007 (2)	0.0025 (17)	0.000 (2)
C26A	0.052 (7)	0.092 (14)	0.028 (6)	0.015 (8)	0.005 (5)	0.001 (7)
C27A	0.064 (8)	0.21 (3)	0.052 (10)	0.042 (14)	0.020 (7)	0.011 (14)
C26B	0.098 (17)	0.079 (19)	0.076 (15)	0.007 (14)	0.001 (11)	−0.022 (13)
C27B	0.087 (19)	0.099 (17)	0.049 (13)	0.010 (13)	0.004 (12)	−0.003 (10)
C28	0.062 (3)	0.066 (4)	0.054 (3)	0.009 (3)	0.021 (3)	0.004 (3)
Cl1	0.0489 (8)	0.0974 (14)	0.1159 (15)	0.0029 (8)	0.0069 (8)	0.0215 (11)
Cl2	0.0906 (13)	0.1116 (16)	0.0762 (12)	0.0305 (11)	0.0160 (9)	0.0255 (10)
Cl3	0.0649 (10)	0.0747 (11)	0.1271 (17)	0.0010 (8)	0.0295 (10)	0.0133 (10)

### Geometric parameters (Å, º)

**Table:**

N1—O2	1.470 (5)	C17—C18	1.524 (8)
N1—C14	1.477 (6)	C17—H17A	0.9800
N1—C5	1.488 (5)	O17—C20	1.428 (6)
O2—C3	1.352 (6)	C18—O18	1.418 (6)
C3—O3	1.203 (6)	C18—C19	1.521 (7)
C3—C4	1.493 (7)	C18—H18A	0.9800
C4—C5	1.531 (7)	O18—C20	1.426 (7)
C4—H4A	0.9700	C19—O19	1.430 (6)
C4—H4B	0.9700	C19—H19A	0.9800
C5—C6	1.512 (6)	O19—H19	0.848 (14)
C5—H5A	0.9800	C20—C22	1.502 (9)
C6—O7	1.454 (5)	C20—C21	1.519 (9)
C6—C10	1.513 (6)	C21—H21A	0.9600
C6—H6A	0.9800	C21—H21B	0.9600
O7—C8	1.428 (6)	C21—H21C	0.9600
C8—O8	1.395 (6)	C22—H22A	0.9600
C8—C9	1.536 (6)	C22—H22B	0.9600
C8—H8A	0.9800	C22—H22C	0.9600
O8—C11	1.427 (7)	C23—C24	1.510 (8)
C9—O9	1.415 (6)	C23—H23A	0.9700
C9—C10	1.535 (6)	C23—H23B	0.9700
C9—H9A	0.9800	C24—O24	1.199 (7)
O9—C11	1.425 (6)	C24—O25	1.322 (7)
C10—O10	1.415 (6)	O25—C26B	1.41 (3)
C10—H10A	0.9800	O25—C26A	1.510 (17)
O10—H10	0.846 (14)	C26A—C27A	1.51 (3)
C11—C12	1.497 (10)	C26A—H26A	0.9700
C11—C13	1.513 (11)	C26A—H26B	0.9700
C12—H12A	0.9600	C27A—H27A	0.9600
C12—H12B	0.9600	C27A—H27B	0.9600
C12—H12C	0.9600	C27A—H27C	0.9600
C13—H13A	0.9600	C26B—C27B	1.43 (4)
C13—H13B	0.9600	C26B—H26C	0.9700
C13—H13C	0.9600	C26B—H26D	0.9700
C14—C15	1.523 (6)	C27B—H27D	0.9600
C14—C23	1.532 (6)	C27B—H27E	0.9600
C14—H14A	0.9800	C27B—H27F	0.9600
C15—O16	1.436 (5)	C28—Cl1	1.741 (6)
C15—C19	1.533 (7)	C28—Cl3	1.747 (7)
C15—H15A	0.9800	C28—Cl2	1.750 (6)
O16—C17	1.410 (6)	C28—H28A	0.9800
C17—O17	1.400 (7)		
			
O2—N1—C14	107.2 (3)	O17—C17—C18	105.8 (4)
O2—N1—C5	105.5 (3)	O16—C17—C18	106.5 (4)
C14—N1—C5	118.0 (3)	O17—C17—H17A	110.8
C3—O2—N1	111.0 (3)	O16—C17—H17A	110.8
O3—C3—O2	119.3 (5)	C18—C17—H17A	110.8
O3—C3—C4	130.1 (5)	C17—O17—C20	110.2 (4)
O2—C3—C4	110.6 (4)	O18—C18—C19	108.8 (4)
C3—C4—C5	103.6 (4)	O18—C18—C17	103.7 (4)
C3—C4—H4A	111.0	C19—C18—C17	104.7 (4)
C5—C4—H4A	111.0	O18—C18—H18A	113.0
C3—C4—H4B	111.0	C19—C18—H18A	113.0
C5—C4—H4B	111.0	C17—C18—H18A	113.0
H4A—C4—H4B	109.0	C18—O18—C20	108.8 (4)
N1—C5—C6	105.2 (3)	O19—C19—C18	108.6 (4)
N1—C5—C4	105.5 (3)	O19—C19—C15	110.4 (4)
C6—C5—C4	116.9 (4)	C18—C19—C15	99.5 (4)
N1—C5—H5A	109.6	O19—C19—H19A	112.5
C6—C5—H5A	109.6	C18—C19—H19A	112.5
C4—C5—H5A	109.6	C15—C19—H19A	112.5
O7—C6—C5	110.2 (3)	C19—O19—H19	107 (5)
O7—C6—C10	103.1 (3)	O18—C20—O17	105.4 (4)
C5—C6—C10	120.0 (3)	O18—C20—C22	108.6 (5)
O7—C6—H6A	107.7	O17—C20—C22	109.2 (5)
C5—C6—H6A	107.7	O18—C20—C21	110.8 (5)
C10—C6—H6A	107.7	O17—C20—C21	108.0 (5)
C8—O7—C6	108.8 (3)	C22—C20—C21	114.6 (6)
O8—C8—O7	111.6 (4)	C20—C21—H21A	109.5
O8—C8—C9	104.9 (4)	C20—C21—H21B	109.5
O7—C8—C9	106.6 (3)	H21A—C21—H21B	109.5
O8—C8—H8A	111.2	C20—C21—H21C	109.5
O7—C8—H8A	111.2	H21A—C21—H21C	109.5
C9—C8—H8A	111.2	H21B—C21—H21C	109.5
C8—O8—C11	111.3 (4)	C20—C22—H22A	109.5
O9—C9—C10	110.0 (4)	C20—C22—H22B	109.5
O9—C9—C8	104.1 (4)	H22A—C22—H22B	109.5
C10—C9—C8	103.3 (4)	C20—C22—H22C	109.5
O9—C9—H9A	112.9	H22A—C22—H22C	109.5
C10—C9—H9A	112.9	H22B—C22—H22C	109.5
C8—C9—H9A	112.9	C24—C23—C14	111.2 (4)
C9—O9—C11	109.4 (4)	C24—C23—H23A	109.4
O10—C10—C6	111.1 (3)	C14—C23—H23A	109.4
O10—C10—C9	109.2 (4)	C24—C23—H23B	109.4
C6—C10—C9	101.4 (3)	C14—C23—H23B	109.4
O10—C10—H10A	111.6	H23A—C23—H23B	108.0
C6—C10—H10A	111.6	O24—C24—O25	124.4 (5)
C9—C10—H10A	111.6	O24—C24—C23	124.8 (5)
C10—O10—H10	106 (4)	O25—C24—C23	110.8 (5)
O9—C11—O8	105.9 (4)	C24—O25—C26B	128.4 (18)
O9—C11—C12	110.9 (6)	C24—O25—C26A	111.8 (8)
O8—C11—C12	108.2 (6)	C27A—C26A—O25	107.4 (18)
O9—C11—C13	108.1 (6)	C27A—C26A—H26A	110.2
O8—C11—C13	108.6 (5)	O25—C26A—H26A	110.2
C12—C11—C13	114.8 (7)	C27A—C26A—H26B	110.2
C11—C12—H12A	109.5	O25—C26A—H26B	110.2
C11—C12—H12B	109.5	H26A—C26A—H26B	108.5
H12A—C12—H12B	109.5	C26A—C27A—H27A	109.5
C11—C12—H12C	109.5	C26A—C27A—H27B	109.5
H12A—C12—H12C	109.5	H27A—C27A—H27B	109.5
H12B—C12—H12C	109.5	C26A—C27A—H27C	109.5
C11—C13—H13A	109.5	H27A—C27A—H27C	109.5
C11—C13—H13B	109.5	H27B—C27A—H27C	109.5
H13A—C13—H13B	109.5	O25—C26B—C27B	105 (2)
C11—C13—H13C	109.5	O25—C26B—H26C	110.7
H13A—C13—H13C	109.5	C27B—C26B—H26C	110.7
H13B—C13—H13C	109.5	O25—C26B—H26D	110.7
N1—C14—C15	115.7 (3)	C27B—C26B—H26D	110.7
N1—C14—C23	106.9 (4)	H26C—C26B—H26D	108.8
C15—C14—C23	110.5 (4)	C26B—C27B—H27D	109.5
N1—C14—H14A	107.8	C26B—C27B—H27E	109.5
C15—C14—H14A	107.8	H27D—C27B—H27E	109.5
C23—C14—H14A	107.8	C26B—C27B—H27F	109.5
O16—C15—C14	109.5 (3)	H27D—C27B—H27F	109.5
O16—C15—C19	103.1 (4)	H27E—C27B—H27F	109.5
C14—C15—C19	116.8 (4)	Cl1—C28—Cl3	109.3 (4)
O16—C15—H15A	109.0	Cl1—C28—Cl2	110.9 (3)
C14—C15—H15A	109.0	Cl3—C28—Cl2	111.9 (4)
C19—C15—H15A	109.0	Cl1—C28—H28A	108.2
C17—O16—C15	108.1 (3)	Cl3—C28—H28A	108.2
O17—C17—O16	111.9 (4)	Cl2—C28—H28A	108.2
			
C14—N1—O2—C3	−113.8 (4)	C5—N1—C14—C15	−65.8 (5)
C5—N1—O2—C3	12.8 (4)	O2—N1—C14—C23	−70.7 (4)
N1—O2—C3—O3	178.7 (5)	C5—N1—C14—C23	170.6 (4)
N1—O2—C3—C4	−0.8 (5)	N1—C14—C15—O16	69.4 (5)
O3—C3—C4—C5	169.4 (5)	C23—C14—C15—O16	−168.9 (4)
O2—C3—C4—C5	−11.0 (5)	N1—C14—C15—C19	−173.9 (4)
O2—N1—C5—C6	105.2 (3)	C23—C14—C15—C19	−52.3 (5)
C14—N1—C5—C6	−135.1 (4)	C14—C15—O16—C17	163.1 (4)
O2—N1—C5—C4	−18.9 (4)	C19—C15—O16—C17	38.1 (5)
C14—N1—C5—C4	100.7 (4)	C15—O16—C17—O17	97.5 (5)
C3—C4—C5—N1	18.2 (4)	C15—O16—C17—C18	−17.7 (5)
C3—C4—C5—C6	−98.3 (4)	O16—C17—O17—C20	−115.1 (5)
N1—C5—C6—O7	−171.3 (3)	C18—C17—O17—C20	0.5 (5)
C4—C5—C6—O7	−54.6 (5)	O17—C17—C18—O18	−15.0 (5)
N1—C5—C6—C10	−51.9 (5)	O16—C17—C18—O18	104.2 (4)
C4—C5—C6—C10	64.7 (5)	O17—C17—C18—C19	−129.1 (4)
C5—C6—O7—C8	163.3 (3)	O16—C17—C18—C19	−9.9 (5)
C10—C6—O7—C8	34.1 (4)	C19—C18—O18—C20	135.3 (4)
C6—O7—C8—O8	101.4 (4)	C17—C18—O18—C20	24.3 (5)
C6—O7—C8—C9	−12.6 (5)	O18—C18—C19—O19	165.1 (4)
O7—C8—O8—C11	−108.6 (5)	C17—C18—C19—O19	−84.5 (5)
C9—C8—O8—C11	6.4 (6)	O18—C18—C19—C15	−79.5 (4)
O8—C8—C9—O9	−17.0 (5)	C17—C18—C19—C15	30.9 (5)
O7—C8—C9—O9	101.5 (4)	O16—C15—C19—O19	72.3 (5)
O8—C8—C9—C10	−131.9 (4)	C14—C15—C19—O19	−47.8 (5)
O7—C8—C9—C10	−13.5 (5)	O16—C15—C19—C18	−41.7 (4)
C10—C9—O9—C11	131.8 (4)	C14—C15—C19—C18	−161.8 (4)
C8—C9—O9—C11	21.7 (5)	C18—O18—C20—O17	−24.5 (5)
O7—C6—C10—O10	75.1 (4)	C18—O18—C20—C22	−141.3 (5)
C5—C6—C10—O10	−47.8 (5)	C18—O18—C20—C21	92.1 (6)
O7—C6—C10—C9	−40.8 (4)	C17—O17—C20—O18	14.2 (6)
C5—C6—C10—C9	−163.7 (4)	C17—O17—C20—C22	130.7 (5)
O9—C9—C10—O10	165.0 (3)	C17—O17—C20—C21	−104.2 (6)
C8—C9—C10—O10	−84.4 (4)	N1—C14—C23—C24	−44.9 (6)
O9—C9—C10—C6	−77.7 (4)	C15—C14—C23—C24	−171.5 (4)
C8—C9—C10—C6	32.9 (4)	C14—C23—C24—O24	118.4 (7)
C9—O9—C11—O8	−18.2 (6)	C14—C23—C24—O25	−60.8 (6)
C9—O9—C11—C12	98.9 (7)	O24—C24—O25—C26B	15 (2)
C9—O9—C11—C13	−134.4 (5)	C23—C24—O25—C26B	−166 (2)
C8—O8—C11—O9	6.6 (6)	O24—C24—O25—C26A	−12.2 (14)
C8—O8—C11—C12	−112.3 (7)	C23—C24—O25—C26A	167.0 (12)
C8—O8—C11—C13	122.5 (6)	C24—O25—C26A—C27A	−174 (3)
O2—N1—C14—C15	52.9 (4)	C24—O25—C26B—C27B	−111 (3)

### Hydrogen-bond geometry (Å, º)

**Table:**

*D*—H···*A*	*D*—H	H···*A*	*D*···*A*	*D*—H···*A*
C4—H4*B*···O16	0.97	2.52	3.083 (6)	117
C5—H5*A*···O2^i^	0.98	2.60	3.469 (5)	148
C8—H8*A*···O19^ii^	0.98	2.62	3.246 (6)	122
O10—H10···O7^iii^	0.85 (1)	1.94 (2)	2.778 (4)	169 (6)
O19—H19···O10^iv^	0.85 (1)	1.98 (3)	2.798 (5)	162 (7)
C28—H28*A*···O3	0.98	2.33	3.172 (7)	144

Symmetry codes: (i) *x*−1, *y*, *z*; (ii) *x*, *y*−1, *z*; (iii) *x*+1, *y*, *z*; (iv) *x*, *y*+1, *z*.
